# Dental profile of a community of recovering drug addicts: Biomedical aspects. Retrospective cohort study

**DOI:** 10.4317/medoral.18669

**Published:** 2013-05-31

**Authors:** María V. Mateos-Moreno, Jaime del-Río-Highsmith, Rafael Riobóo-García, Maria F. Solá- Ruiz, Alicia Celemín-Viñuela

**Affiliations:** 1 Department of Stomatology IV. Faculty of Dentistry. Complutense University of Madrid, Madrid, Spain; 2 Department of Stomatology I. Faculty of Dentistry. Complutense University of Madrid, Madrid, Spain; 3 Department of Prosthodontics. Faculty of Medicine and Dentistry. Valencia University, Valencia, Spain

## Abstract

Objectives: to obtain a biomedical oral profile of a community of adult drug addicts in treatment by analysing their dental health, with a view to determining whether the state of their oral health could be attributed primarily to their lifestyle and the direct consequences of drug abuse on their overall condition, rather than to the effects of the drugs used. Experimental Design: the study was conducted under the terms of an agreement between the Complutense University of Madrid’s (UCM) Odontology Faculty and the City of Madrid’s Substance Abuse Institute. Seventy drug addicts and 34 control group subjects were examined. The study assessed oral hygiene habits, systemic pathology, type of drugs used and the duration of use, oral pathology, oral health indices, risk of caries based on saliva tests, oral candidiasis and periodontal microbiology. Results: statistically significant differences (p<0.05) were found between the test and control groups for practically all the variables analysed. In the drug users group, dental hygiene was wanting, systemic and oral pathology prevailed and the decayed/missing/filled teeth or surface (DMFT/S) indices denoted very poor buccodental health. The saliva tests showed a substantial risk of caries and candidiasis rates were high. By contrast, with a single exception, the microbiological studies detected no statistically significant difference between drug users and control groups periodontal flora. Conclusions: drug-dependent patients had poor oral health and a significant increase in oral pathology, essentially caries and periodontal disease. Their risk of caries was high and the presence of candidiasis was representative of their poor general and oral health. Drug users’ poor buccodental condition was more closely related to lifestyle than to drug abuse itself.

** Key words:**Buccal, dental, drug addicts.

## Introduction

In 1992, the WHO defined drug addiction as “a state, psychic and sometimes also physical, resulting in the interaction between a living organism and a drug, characterized by behavioural and other responses that always include a compulsion to take the drug on a continuous or periodic basis in order to experience its psychic effects, and sometimes to avoid the discomfort of its absence. Tolerance may or may not be present.”

Some of the most prominent conclusions on substance abuse trends and problems in Spain in recent years include the following. Tobacco use, observed to decline through 2007, has since flattened; the proportion of alcohol users is steady or declining, although the rate of intensive consumption episodes is rising; the number of intravenous drug users receiving treatment is no longer contracting; mortality directly related to illegal drugs is gradually falling; high HIV and hepatitis infection rates persist in I/V drug users, along with risky sex and injection behaviour, although the number of new HIV diagnoses has dropped steeply; heroin use may be flattening or even rising after many years of decline; cocaine and cannabis use has stabilised or may have begun to slide; hypnosedative drug use is rising, while the use and abuse of ecstasy, amphetamines and hallucinogens continues to fall.

Very little research today addresses drug users’ oral health, among other reasons because of the difficulty inherent in monitoring and tracking the subjects (1,2). One pioneer study noted that drug users tend to suffer from anxiety in connection with dental treatment and report difficulties in accessing such care, or even reluctance among some dentists (as well as other health care professionals) to treat them (3,4). As their pain tolerance is generally low, relief must be administered carefully and good rapport with the dentist established (5). As a rule they are scantly motivated to abide by oral health standards or guidelines, or to follow treatment recommendations. Dentists should be alert to and able to identify drug abuse-related oral problems (6) to furnish suitable information and care (4). Dental treatment needs to be systematically included in the comprehensive treatment afforded drug users (7-10). Moreover, the treatment plan should be personalised (11). An accurate stomatological assessment is a must for these patients, who need increasingly frequent dental care (12). The present study was conducted in the context of the dental care provided as one of a series of measures to further social reinsertion. Implicit in this action was the recognition of the importance of psychological balance, which is largely dependent on physical appearance and quality of oral life. In this high dental risk community, care calls for close collaboration between social work and health care professionals (13,14).

## Material and Methods

1. Material. Reference population and sampling

A retrospective cohort study was conducted under an agreement between the Complutense University of Madrid’s Odontology Faculty and the City of Madrid’s Health Substance Abuse Institute. A total of 70 drug addicts in treatment, ranging in age from 30 to 56, were assessed. Prior to the study, a referral report was received from the Substance Abuse Institute’s Drug User Assistance Centres (Spanish initials, CAD) or Proyecto Hombre, an NGO engaging in drug addition treatment and prevention. All the patients were institutionalised in these centres, from which they were referred to the Odontology Faculty for comprehensive, cost-free dental treatment (funded by the Substance Abuse Institute). Buccodental health was also analysed in a control group of 34 patients, ranging in age from 30 to 59.

The patients were informed of their state of oral health, the objectives of the study and the methods to be used. All voluntarily agreed to participate in the study and signed an informed consent form. All the examinations were conducted by just two dentists. The Kappa intra- and inter-observer diagnostic concurrence values for the oral examinations were never lower than 0.60, i.e., within the suitability range.

2. Method

Patients were examined and treated as part of the undergraduate course entitled Integrated Adult Clinical Dentistry (Stomatology I Department).

2.1. Clinical history: referring facility, reason for the consultation, health questionnaire, drug use history (type of drug used, year of initiation, habituation and discontinuation, administration method, frequency of use in the last year and month), dental history (anxiety around dental treatment, possible allergy to dental anaesthesia, oral hygiene habits and the existence in the past or present of mouth burn, ulcers, foul taste, halitosis, retching, gum bleeding or dry mouth) and lastly, family background.

2.2. Examination: soft tissue (general, locoregional and buccal), dental and periodontal.

Each substance user’s most frequent pathologies were recorded on the grounds of the definitions in the scientific literature: rampant caries, root caries, oral mucositis, angular cheilitis, candidiasis, herpes, ulceration, pappose leukoplakia, gingivitis, periodontitis, papilloma, oral pain and xerostomia.

 Oral health indices were also determined to evaluate the prevalence of caries (DMFT, DMFS and Katz root caries indices) and periodontal disease (Conroy and Sturzenberger severity of surface dental calculus (CSSI), bleeding or gingival index and the O’Leary plaque index).

2.3. Supplementary trials:

a) Photographs.

b) Orthopantomography.

c) Biopsy: only where suspected and in the absence of a clinical diagnosis of the damage observed.

d) Saliva tests:

d.1. Determination of volume: measured after stimulation (normal stimulated salivary flow = 1 2 ml/min).

d.2. Determination of salivary pH: measured in the Odontology Faculty laboratory with a Crison® micropH 2001 pH-meter (nor-mal pH may range from 5.6 to 7.6 (~6.75).

d.3. Determination of buffer capacity: using the CRT® buffer test.

d.4. Streptococcus and Lactobacillus count: measured with the CRT® bacteria technique.

e) Detection of specific bacteria in periodontal pockets: the deepest pocket areas in each quadrant of the buccal cavity were located and samples were taken in four wherever possible, inserting two number 30 sterile paper tips per periodontal pocket. The samples were analysed for the following microorganisms: Aggregatibacter actinomycetemcomitans, Porfiromona gingivalis, Prevotella intermedia, Micromonas micros, Eikenella corrodens, Fusobacterium nucleatum, Tannerella forsythia, Campylobacter rectus, Capnocytophaga spp, Eubacterium spp.

f) Dorsal lingual and jugal mucous membrane smears: taken with sterile cotton swabs and analysed in the laboratory for Candida spp.

2. 4. Statistical analysis: the data were analysed with IBM SPSS Statistics 19.0® software. The first phase of the statistical study consisted of a descriptive analysis of all the variables. That was followed by a comparison of the continuous variables in terms of a categorical variable for the two groups of populations (drug addicts vs control). The statistical tests applied to determine the existence of significant differences between the control and experimental groups were Student’s t-test (comparison of two means, assuming equal variance and normal distributions) and the Welch-adjusted Student’s t-tests (for unequal variance). Two categorical variables were also compared (in per cent) and the p values were obtained with either the Pearson’s chi-square or a likelihood ratio test. Lastly, contingency tables and independence tests (such as chi-square or Fisher) were used to assess the effect of certain variables on the drug user group, along with means, standard deviations and comparison of means (with ANOVA or Student’s t). The value adopted for statistical significance was 0.05.

## Results

The total number of subjects in this group was reduced to 64 poly-drug users, after six alcohol users only were excluded.

Most (89.1%) of these patients had low and the rest (10.9%) medium social-cultural backgrounds. None were from a high social-cultural background.

For 32.8% (n=21), the primary reason for seeking care was to reestablish buccodental function, while 31.3% (n=20) were con-cerned about their health and 29.7% (n=19) about their appearance. Only 6.3% (n=4) of the patients presented with pain. While the drug-dependent patients were more interested in recovering their buccodental function, the control group showed a greater interest in their general health. This difference was statistically significant (p<0.001).

Oral hygiene habits were deficient in 64.1% (n=41) of drug users, who said they never brushed their teeth; 17.2% (n=11) brushed once a day and 18.8% (n=12) twice or more. In this same group, 67.2% (n=43) reported that they used a mouthwash. Statistically significant differences were found between the two groups: most of the control group brushed their teeth more than twice daily (p<0,001) and were more frequent users of supplementary oral hygiene methods such as interdental (p 0.047) or electric (p 0.002) toothbrushes.

The systemic pathology in the experimental group was HCV in 76.6% (n=49), followed by HBV in 62.5% (n=40) and tuberculosis (or contact with tuberculosis) in 54.7% (n=35). HIV infection was present in 39.1% (n=25). Respiratory problems were also common, affecting 37.5% (n=24) of the patients. Traumatism and gastric disorders were likewise prevalent (35.9% and 31.3%, respectively). Overall, 29.7% of the patients had psychiatric/psychological pathologies, and 21.9% (n=14) had a sexually trans-mitted disease. The experimental group had higher rates of tuberculosis (p<0.001), hepatitis C (p<0.001), hepatitis B (p<0.001), HIV (p<0.001), sexually transmitted disease (p 0.016) and psychiatric disorders (p 0.022) than the control group. The incidence of orofacial traumatism was also higher in the former (p 0.016).

A total of 26.6% (n=17) of the experimental group was on antiretroviral medication, 57.8% (n=37) took psychiatric drugs, 17.2% (n=11) medicine for ulcers, 6.3% (n=4) vitamin supplements and 20.3% (n=13) were medicated for other disorders such as epilepsy or chronic alcoholism. Differences were unsurprisingly found between the two groups: consumption of anti-HIV (p<0.001), psychiatric (p<0.001) and other types of medication (p<0.01) was higher among the drug addicts.

Heroin and cocaine were consumed by 96.8% and 90.6% of the members of this group, respectively, and were the drugs most commonly used, together with tobacco (98.4%). Methadone was consumed by 92.18% (n=59), at doses ranging from 5 to 150 mg/day. Alcohol was consumed chronically by 53.1% (n=34), benzodiazepine by 32.8% (n=21) and cannabis by 65.6% (n=42). The heroin addicts had been using the drug for 8 to 30 years, while the patients dependent on cocaine had been users for 6 to 30 years. The ranges for cannabis and tobacco were 8 to 29 and 17 to 40 years, respectively. Alcohol was consumed sporadically by 4.69% (n=3). The duration of use was longer in the drug user group than in the control group for the only two drugs consumed by the latter, tobacco and cannabis (sporadically) (p<0.001).

**Figure 1:**
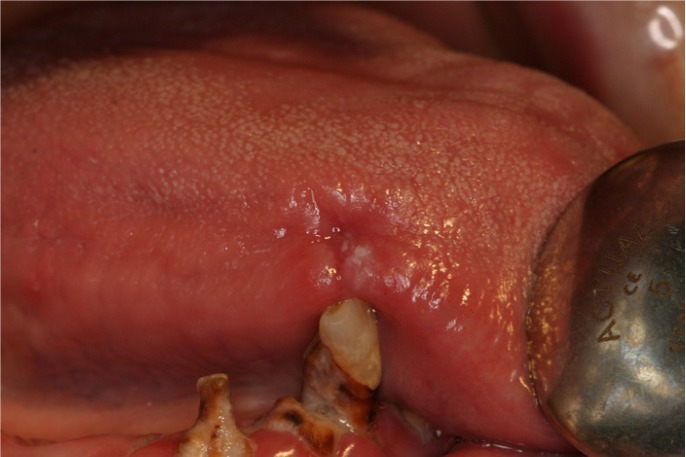
Mouth ulcer.

Xerostomia was perceived by 64.1% (n=41) of the user patients. Retching, halitosis and gum bleeding were reported by 35.9% (n=23), foul taste by 42.2% (n=27) and oral pain by 62.5% (n=40). Five patients, or 7.8%, claimed to have mouth ulcers (Fig. 1) and the same proportion mouth burn. Dental treatment inspired anxiety in nearly half of the patients (45.3%, n=29). With the exception of gum bleeding, which at 64.7% (n=22) was higher in the control group, all these symptoms were more accentuated in the drug-dependent group (p 0.007).

No significant differences were observed between the two groups in connection with adenopathies, although they were more frequent among the experimental group (n=17).

**Figure 2:**
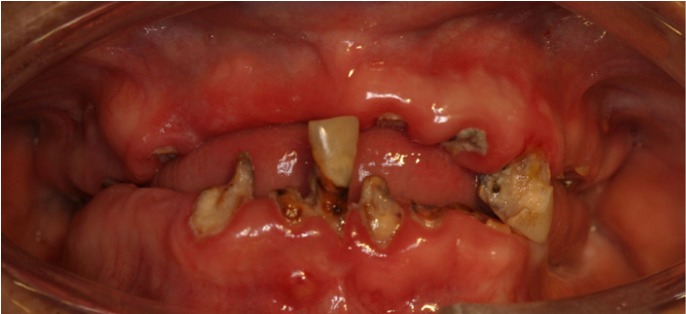
Rampant caries.

The prevailing oral pathology among drug users was periodontitis, with 81.3% (n=52). Half of the patients presented with rampant caries (Fig. 2). At the time of the examination, seven (10.9%) were found to have angular cheilitis. Six (9.4%) had ulcers on the mucous membrane of the oral cavity. Leukoplakia and mucositis were diagnosed in only three patients (4.8%), while herpes was present in only two. Lastly, only one patient (1.6%) had gingivitis and papilloma. Discrepancies between the two groups were found for rampant caries only, whose incidence was higher among the addicts (p<0.001). Among the periodontal disorders, gingivitis was more common in the control group (p<0.001) and periodontitis in substance users (p<0.001) ( Table 1).

**Table 1:**
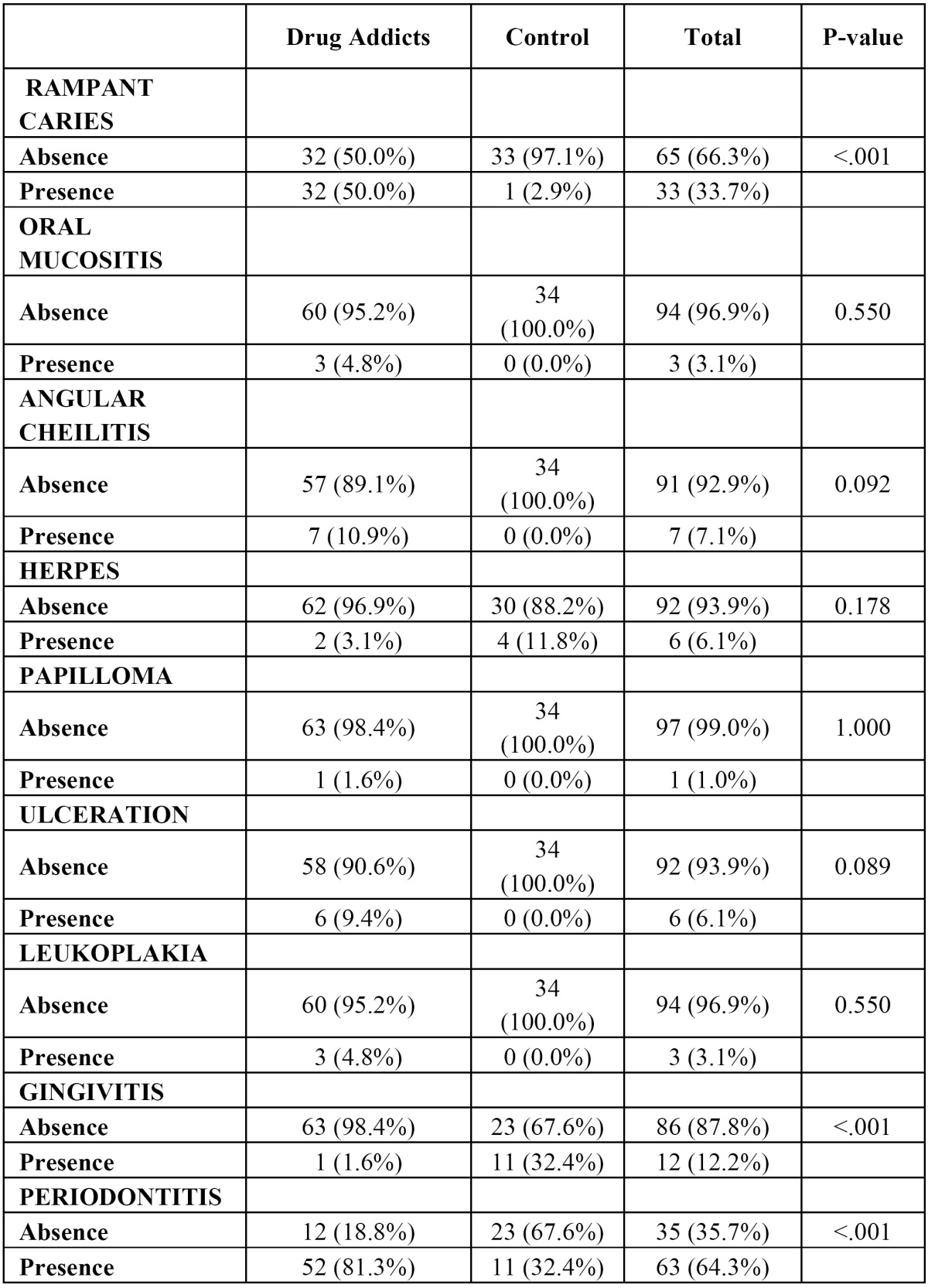
Comparative: Oral pathology. Drug Addicts vs Control.

The DMFT index in the test group was 22.7 (n=60) and the DMFS index 91.1 (n=60). The four toothless patients were excluded from these calculations. The mean indices for decayed (D), missing (M) and filled (F) teeth were 10.1, 12.1 and 0.7, respectively. The root caries index (RCI) was 23.9% on average. The means for the plaque (PLAQUE I.) and gingival (GINGIVAL I.) indices were 74% (n=53) and 56.8% (n=54), respectively. The mean calculus index (CALCULUS I.) was 15.8. Statistically significant differences were found between the two groups in all the indices (p<0.001), with consistently higher values in the test group. The percentage of decayed and missing teeth was also higher among the drug users (p<0.001), while the control group had a higher proportion of filled teeth (p<0.001). The calculus index was the sole measure in which the two groups had similar scores (p 0.992) ( Table 2).

**Table 2:**
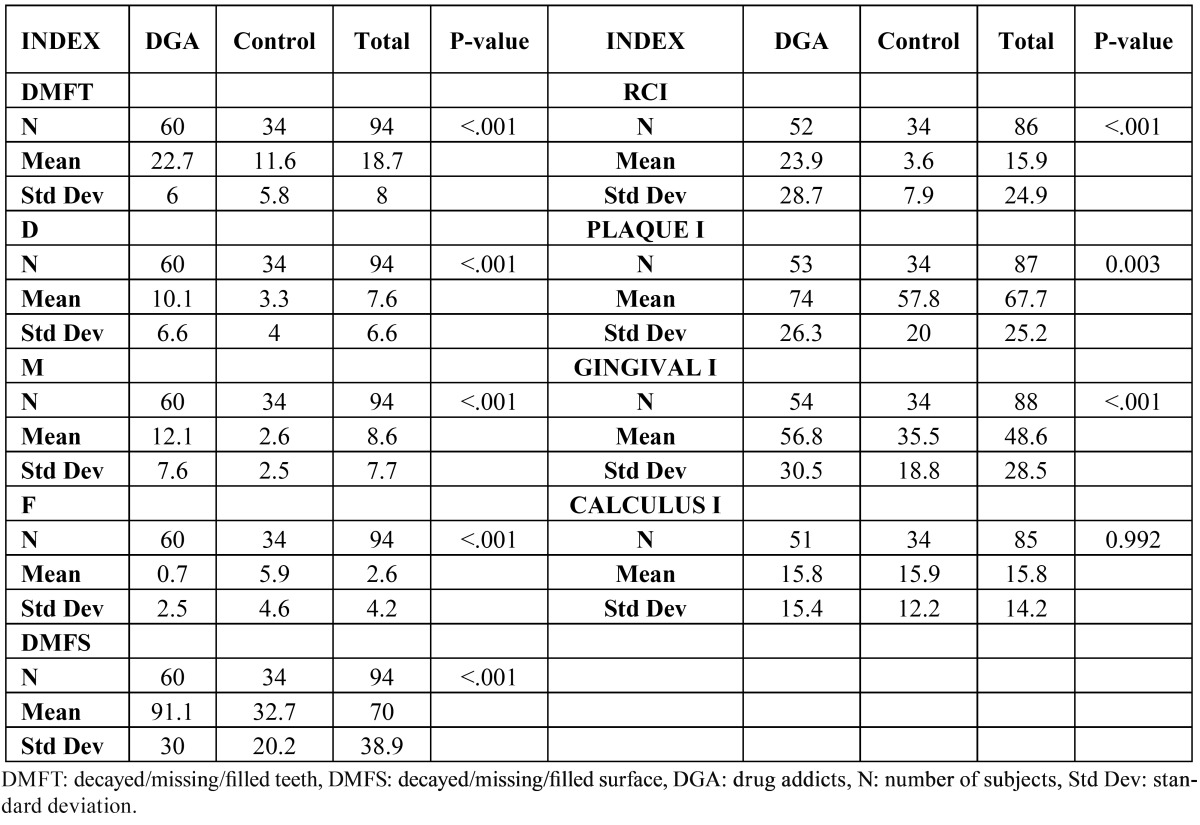
Comparative: Oral health indices. Drug addicts vs Control.

The smears revealed the presence of candida in 78.12% (n=50) of the addict patients, a rate significantly higher than found in the control group (p 0.027).

In the experimental group, the mean stimulated salivary secretion was 4 ml/5 minutes (n=64) (not significantly different from the control, p 0.967). The mean pH in the former, 6.8 (n=58), was slightly slower than for the latter (p<0.01). The buffer capacity was high in 32.8% (n=21) of the user patients, medium in 23.4% (n=15) and low in 43.8% (n=28). Differences were also identified between the two groups with respect to this variable (p<0.001): while low capacity prevailed among the substance users, half of the control group members exhibited high capacity. According to the microbiological findings, 67.3% (n=37) of the users` salivary samples contained over 105 CFU/ml (colony-forming units per millilitre) of Streptococcus mutans, while 81.4% (n=48) contained over 105 CFU/ml of Lactobacillus. The presence of both S. mutans (p 0.044) and Lactobacillus (p<0.001) was higher in the drug addicts group ( Table 3).

**Table 3:**
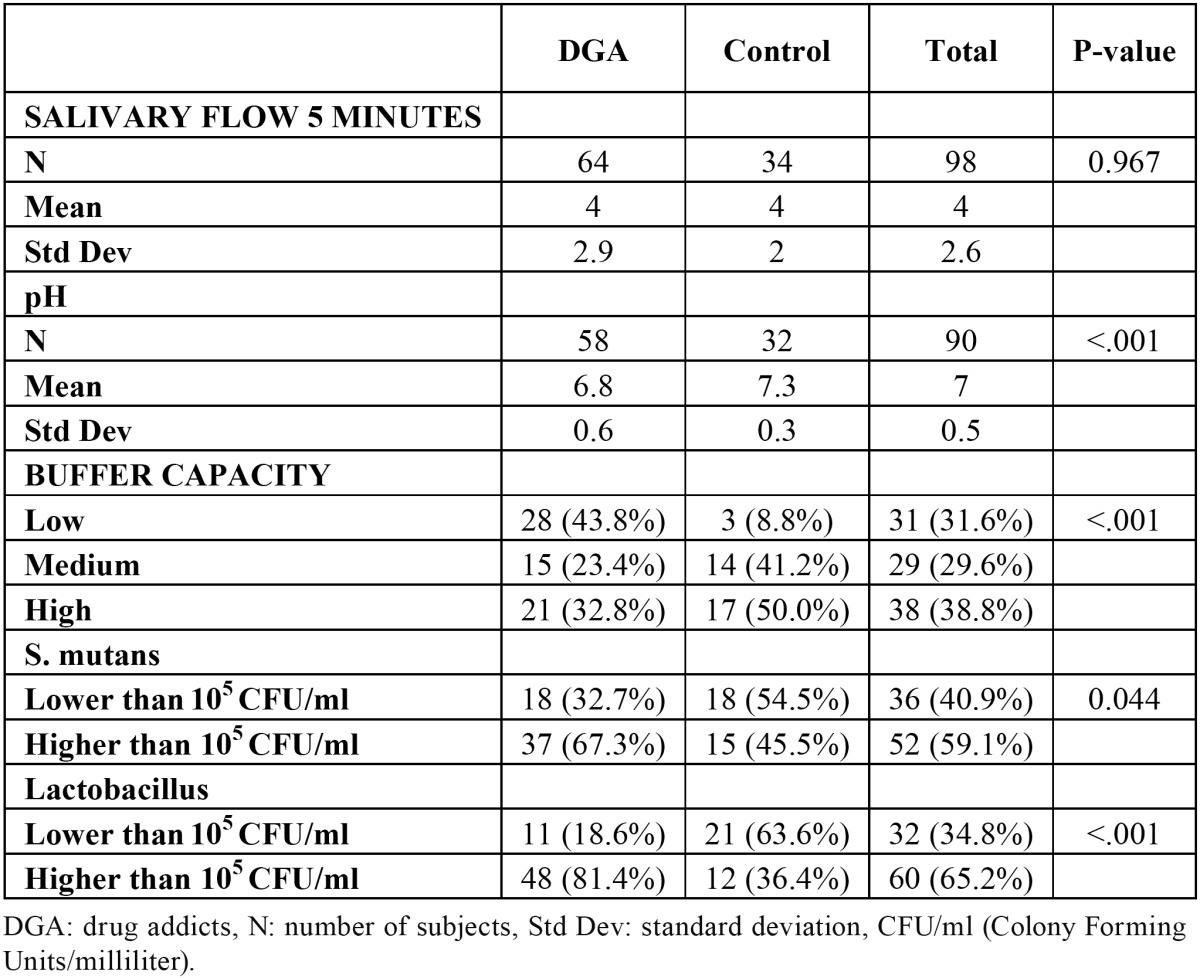
Comparative: Saliva tests. Drug addicts vs Control.

The mean values for the number of patients with periodontal microorganisms are listed in  Table 4, which shows that F. nucleatum, the most prevalent pathogen, was found in 47 of the patients analysed. No statistically significant differences were observed between the two groups in connection with periodontal pathogens, with the exception of higher rates of Eubacterium spp in the control group (p<0.001) and the higher bacterial load in the test group (p 0.027). The mean number of microorganisms detected in each patient, i.e., the total periodontal flora (CFU) was 8 076 568.627 among addicts and 3 079 676.471 in the control group.

**Table 4:**
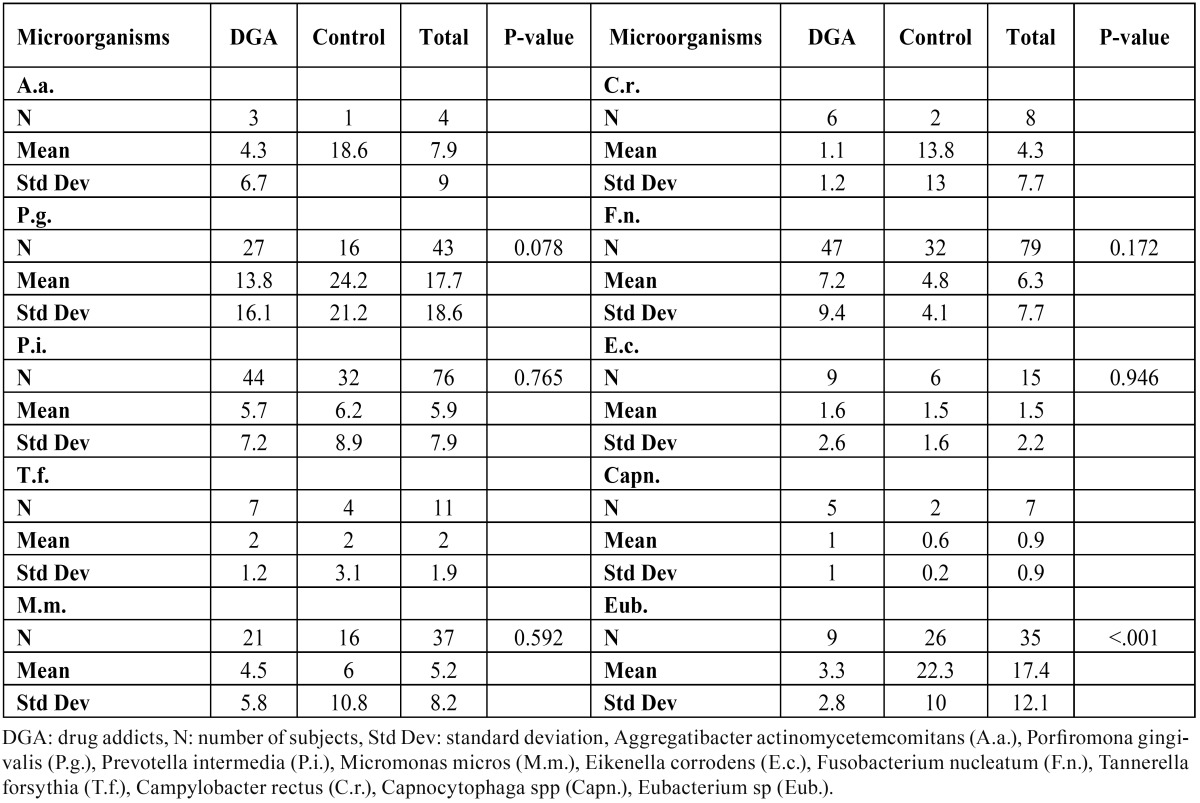
Comparative: Periodontal microbiology. Drug addicts vs Control.

## Discussion

The effects of illegal drug abuse on buccal health has been scantly researched due to the difficulty in marshalling drug-dependent patients. Like the authors of the present study, researchers normally analyse patients in drug rehabilitation centers (15). As a rule, these patients only visit a dentist to solve an incidental problem arising at any given time, but once they have received emergency care, they are unlikely to take the professional advice given or keep follow-up appointments. In the present study, what patients wanted above all was to improve their oral health and hence their overall quality of life.

The present findings respecting poor oral health habits among drug addict patients, attributable essentially to their scant dental health education and low self-esteem, were consistent with earlier reports (16). When using, these patients needed to obtain drugs to avoid painful withdrawal symptoms. Personal hygiene was neither necessary nor important under those circumstances. In a very prominent article in the scientific literature on drug use, Scheutz proved that when methadone is administered during habit-breaking periods, oral hygiene improves considerably (1). The symptoms of caries may on occasion be masked by drugs, except during withdrawal or when an addict is on methadone, which is a weaker pain killer than heroin (16,17).

The present study found that patients with poor oral hygiene had higher caries indices. Moreover, the fact that they had filled teeth denoted a greater interest in their dental health. This is significant, for despite all the problems deriving from drug addiction, if these individuals are persistent in their good dental health habits, their oral cavity will remain healthy. It shows that lifestyle and healthy habits are essential in preventing buccodental pathologies in these patients.

One of the most severe consequences of drug addiction is the appearance of deleterious systemic disease which, together with the associated medication, has a heavy impact on buccal health (18). HIV infection also affects the oral cavity (19), while methadone induces xerostomia and its high sugar content raises the risk of caries (20).

The present observations on the type of drugs used concur with other reports on poly-drug addiction (10). Poly-drug use by all the patients is a problem frequently encountered in research on addiction, and, consequently, is very difficult to study the individual effect of a given drug in such a scenario (21).

The present study also concurs with the literature in that patients identify a given oral pathology over the rest. Even so, patients thought that methadone was the primary cause of their oral deterioration (16).

The damage most frequently found in the scientific literature was as follows: reddened oral mucous membrane (only three, an insignificant number, of the patients in this study presented with mucositis) and pappose tongue (no case was found here). One paper (22) reported 6.6% leukoplakia, a percentage slightly higher than in the present study (4.8%). Studies have been conducted on the impact of each drug separately on the oral cavity, such as the necrotising effect of cocaine on the oral mucous membrane and the consequential appearance of oronasal communication due to ischemic necrosis of the palate (23). Substance addict patients have a significantly high rate of infection-related oral problems due largely to immunodeficiency (24). In the present study, the periodontal problems detected were in an advanced stage and half of the patients exhibited rampant caries. Most scientific papers focus primarily on these two pathologies and report findings that concur with the present results. One example can be found in Dedi´c (9).

The oral health indices found here were compared to the results of an Italian study on poly-drug users (13). There, the DMFT index was 12.9, lower than the present value. In the Italian study, the breakdown of the DMFT components showed that caries accounted for most of the index, denoting a pressing need for treatment. As in the present study, only a small fraction of teeth had fillings, an indication of the lack of patient interest in their oral health and a prevalence of extraction as the treatment of choice. Many other authors have drawn that same conclusion (25). The literature contains very few references to the DMFS index (1,25). Caries are often positioned in the cervical region, frequently involving the root surface. In early research on addiction, that position was in fact described as a pathognomonic symptom of the abuse of certain drugs (26). Quantification of root caries, for which no measure was found in the literature, is an area in need of further research.

Like the present study, the literature consistently reports high plaque, gingivitis and bleeding indices among drug addicts (1,27). The figures on gum bleeding are more scattered, or in some cases fail to specify whether bleeding was spontaneous or subsequent to periodontal probing. In previous studies, such as by Du et al. (27), the presence or absence of calculus was mentioned, but with no information on amount or position.

The present results on the risk of caries and periodontal disease in drug-dependent patients could not be compared to previous findings. Not a single study was found that systematically measured salivary flow, pH or the buffer capacity of saliva (although references were found to the xerostomising effect of medication, HIV patients, and based simply on patient perception of dry mouth) (26,28) or conducted tests to determine the presence of cariogenic microorganisms, such as Streptococcus mutans and Lactobacillus (here Lactobacillus concentration was found to be significantly higher in the drug addict group, denoting much more intense caries activity.) Much the same is true of the determination of periodontal flora (studied only in HIV patients, addicted in some cases to drugs administered parenterally) (29).

The present findings are consistent with other reports to the effect that a series of factors stemming from drug addiction are asso-ciated with high Candida levels in the body (30).

The conclusion that can be drawn from the foregoing is that drug users in treatment had a significantly high rate of oral pathology, essentially caries and periodontal disease. Their risk of caries was high and the presence of candidiasis was representative of these patients’ poor general and oral health. Drug abuse for long periods of time and their local and systemic effects are only one of the many factors that cause this group’s oral problems. The combination of systemic immunodeficiency, severe organic pathology and associated medication, and a series of social factors such as unemployment and social exclusion, may be regarded as direct causes of the deterioration of oral health. In addition, a cariogenic diet, scant oral hygiene, low self-esteem and little or no perception of health issues, not to mention very limited accessibility to dental services, also contribute to the development of oral pathologies. Drug addiction and concomitant psychic deterioration induce neglect of oral hygiene, which is the primary cause of deleterious changes in the oral cavity. The present study shows that this community of patients needs special dental care and that dentists play a key role in drug use prevention and control. Policies that enhance access by drug addicts and other marginal groups to dental services are much needed.
